# The strong focus on positive results in abstracts may cause bias in systematic reviews: a case study on abstract reporting bias

**DOI:** 10.1186/s13643-019-1082-9

**Published:** 2019-07-17

**Authors:** Bram Duyx, Gerard M. H. Swaen, Miriam J. E. Urlings, Lex M. Bouter, Maurice P. Zeegers

**Affiliations:** 1 Nutrition and Translational Research in Metabolism (School NUTRIM), Maastricht University, Maastricht, The Netherlands; 2 Department of Epidemiology and Biostatistics, Amsterdam University Medical Centers, Amsterdam, The Netherlands; 3 Department of Philosophy, Faculty of Humanities, Vrije Universiteit, Amsterdam, The Netherlands; 4 Care and Public Health Research Institute (School CAPHRI), Maastricht University, Maastricht, The Netherlands

**Keywords:** Reporting bias, Systematic reviews, Search engines, Bladder cancer, Epidemiology, Diesel exhaust exposure, Abstract, Keywords

## Abstract

**Background:**

Research articles tend to focus on positive findings in their abstract, especially if multiple outcomes have been studied. At the same time, search queries in databases are generally limited to the abstract, title and keywords fields of an article. Negative findings are therefore less likely to be detected by systematic searches and to appear in systematic reviews. We aim to assess the occurrence of this ‘abstract reporting bias’ and quantify its impact in the literature on the association between diesel exhaust exposure (DEE) and bladder cancer.

**Methods:**

We set up a broad search query related to DEE and cancer in general. Full-texts of the articles identified in the search output were manually scanned. Articles were included if they reported, anywhere in the full-text, the association between DEE and bladder cancer. We assume that the use of a broad search query and manual full-text scanning allowed us to catch all the relevant articles, including those in which bladder cancer was not mentioned in the abstract, title or keywords.

**Results:**

We identified 28 articles. Only 12 of these (43%) had mentioned bladder in their abstract, title or keywords. A meta-analysis based on these 12 detectable articles yielded a pooled risk estimate of 1.10 (95% confidence interval [CI] 0.97–1.25), whereas the meta-analysis based on all 28 articles yielded a pooled estimate of 1.03 (95% CI 0.96–1.11).

**Conclusions:**

This case study on abstract reporting bias shows that (a) more than half of all relevant articles were missed by a conventional search query and (b) this led to an overestimation of the pooled effect. Detection of articles will be improved if all studied exposure and outcome variables are reported in the keywords. The restriction on the maximum number of keywords should be lifted.

**Electronic supplementary material:**

The online version of this article (10.1186/s13643-019-1082-9) contains supplementary material, which is available to authorized users.

## Background

Systematic reviews and meta-analyses play a key role in science. They are considered to provide the highest level of evidence (e.g. [1, 2]). To some extent, they also provide a correction mechanism for distorting phenomena such as citation bias [3] or small sample size bias [4]. In addition, by summarising all available evidence, they reach higher precision and enable exploration of heterogeneity between studies.

It is crucial that systematic reviews capture the totality of the available evidence and not a biassed subset. For this, a proper literature search methodology is essential. We already know that negative findings do not always get published due to publication bias (e.g. [5]) and outcome reporting bias [6]. This makes them less likely to be included in systematic reviews. However, even if negative findings get published, they might still be overlooked by systematic reviews due to *abstract reporting bias*. This is the phenomenon that negative findings are less likely to be reported in abstracts than positive findings, even if they are conscientiously reported in the full-text of a publication. We are aware of one previous article that observed biassed reporting in abstracts [7]. However, to the best of our knowledge, the idea that abstract reporting bias may distort systematic reviews has never been proposed or studied before.

Systematic reviews rely on a systematic search in databases such as Web of Science and PubMed. By default, however, these searches are limited to the title, abstract and keywords of an article. Full-texts are not searchable in these general databases [8, 9]. Therefore, if results are only reported in the full-text of an article, and not in the abstract, title or keywords, they will be missed by current search methods. In addition to the author keywords, database providers can create their own keywords, such as Medical Subject Headings (MeSH). These database-specific keywords are also searchable and potentially improve the completeness of a search.

The systematic review method has been extensively used in clinical medicine. Because of its great merits, the method is now also applied to observational studies. However, there are profound differences between clinical trials and observational studies. Clinical trials often focus on a single intervention and a single primary outcome. Observational epidemiology studies generally investigate multiple associations. A single risk factor can be correlated with numerous outcome parameters. This is particularly the case in cohort studies.

If multiple health outcomes are investigated and reported in the full-text of an article, but not all of these outcomes are reported in the abstract, then a systematic search may not catch all relevant studies. It seems likely that positive results are more prone to be reported in the abstract. If this is indeed the case, then this would lead to an underrepresentation of negative results in systematic reviews and to an overestimation of the pooled effect size in meta-analyses.

In the current study, we investigated the occurrence and impact of abstract reporting bias for a specific research topic: the association between diesel exhaust exposure (DEE) and bladder cancer. This topic was chosen because we anticipated that a substantial number of studies on this association would have a cohort design allowing to investigate multiple health outcomes simultaneously.

## Method

We performed a case study of abstract reporting bias in the empirical literature investigating the association between DEE and bladder cancer. The protocol of this study, including the data-analysis plan, was pre-registered on DataVerse [10]. All analyses were pre-planned unless stated otherwise. Deviations from the protocol are described in Additional file 1: Text S1.

### Search strategy and article selection

We set up a broad search strategy in order to identify all the relevant articles on this topic, including those that did not mention bladder cancer in their abstract, title or keywords. We therefore did not restrict our query to bladder cancer, but focused on cancer in general (Table 1). The query was applied to Web of Science Core Collection (WoSCC) and to PubMed.

**Table 1:** Search strategy in Web of Science and PubMed

Database/search engine	Web of Science Core Collection (WoSCC)	PubMed
Broad search query	((diesel) and (exhaust or particulate matter or microparticles or emissions or exposure)) AND (cancer or carcino* or neoplasm or tumo*))	(((diesel) and (exhaust or particulate matter or microparticles or emissions or exposure)) AND (cancer or carcinogen* or carcinoma or carcinomas or carcinomous or carcinoid or carcinoids or neoplasm or tumor or tumour or tumorous or tumors or tumours)) AND (Humans[Mesh])
Specific search query (restricted to bladder cancer)	((diesel) and (exhaust or particulate matter or microparticles or emissions or exposure)) AND (cancer or carcino* or neoplasm or tumo*)) *AND(bladder or urinary or transitional)*	(((diesel) and (exhaust or particulate matter or microparticles or emissions or exposure)) AND (cancer or carcinogen* or carcinoma or carcinomas or carcinomous or carcinoid or carcinoids or neoplasm or tumor or tumour or tumorous or tumors or tumours)) AND (Humans[Mesh]) *AND (bladder or urinary or transitional)*
Search fields	‘Topic’ (abstract, title, author keywords, KeyWords Plus)	‘All fields’ (including abstract, title, author keywords, MeSH terms)
Database-specific keywords	KeyWords Plus, added by Clarivate Analytics based on each article’s reference list [12]	MeSH terms, suggested by an algorithm based on the full-text of each article and manually checked by professional indexers from the U.S. National Library of Medicine [13]
Search date	16 September 2018	16 September 2018

Notes: Applying the WoSCC search query including wildcards to PubMed led to an error message stating that there were too many references that could not all be shown. Therefore we made some minor adaptations to the query in PubMed. The PubMed search is automatically expanded to include relevant MeSH terms. The *broad search query* was used to identify all the relevant articles for our study sample. The *specific search query* was used to check the assumption that it would fail to identify articles with incomplete abstract reporting

**Fig. 1:**
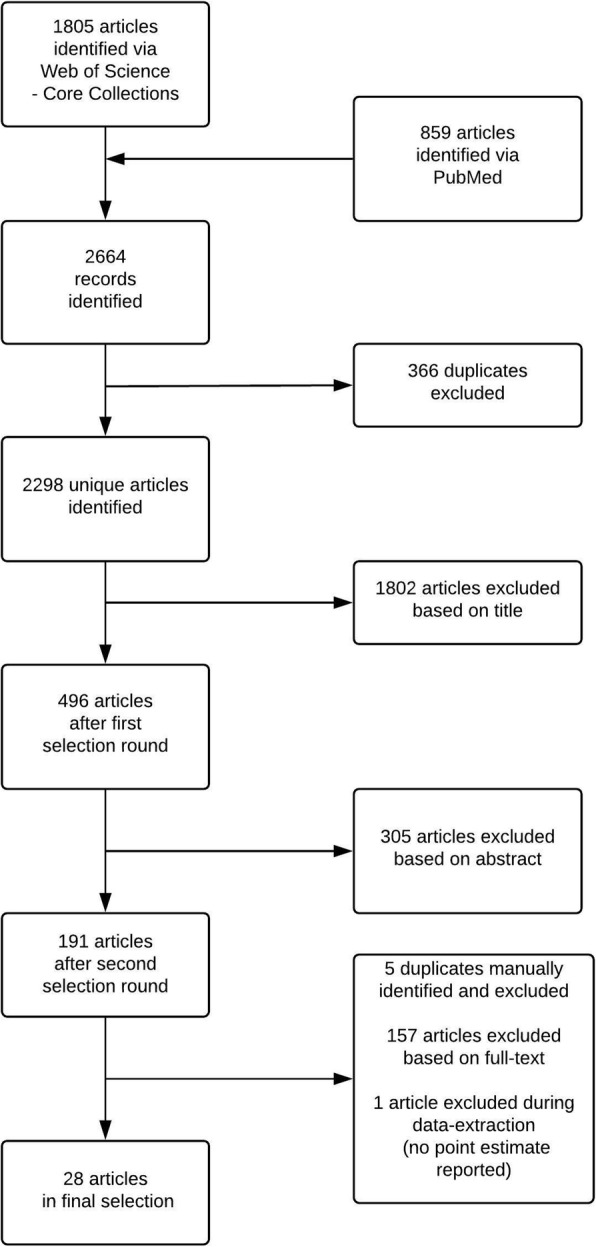
Flow diagram of article selection process (based on broad search query)

Article selection was performed in three steps (first based on title, then on abstract and finally on full-text; Fig. 1). The first two steps were done conservatively; if there was any suggestion of multiple health outcomes, the study would be selected for the next step. In the third step, all full-texts were read to decide whether bladder cancer was one of the health outcomes studied. Articles were selected by two independent assessors (BD and GMHS). Results were compared after the last step, and consensus was always reached.

All English-language cohort studies reporting results on the association between DEE and bladder cancer were included. This includes studies that defined DEE by means of job occupations with a high exposure to diesel exhaust. Only cohort studies were included, as they are most likely to report multiple health outcomes. Studies in which bladder cancer was not mentioned as a separate health outcome, but classified in the same outcome category as other urinary organs (such as in the ICD-7 classification for urinary tract) and kidney, were also included. Studies in which bladder cancer was classified in the same outcome category as prostate cancer (which has a much higher prevalence) were excluded.

The included articles formed our study sample. We assumed to have captured all relevant articles due to our broad search strategy on cancer in general. After all, if bladder cancer is the only health outcome studied, it should be reported as such in the abstract. If it is not the only health outcome, then it is most likely that other types of cancer were tested as well. In other words, we believe that the word cancer or one of its synonyms would always be reported in the abstract or keywords of studies that include bladder cancer as a health outcome.

### Data extraction

Data extraction was performed by two independent assessors (BD and GMHS), followed by a consensus meeting.

We extracted the *point estimate* and *95% confidence interval* of the unadjusted effect size that was reported in the articles. In most articles, the reported effect size concerned a standardised mortality ratio, but it could also be a standardised incidence ratio, a risk ratio or a proportional mortality ratio. If no confidence interval had been reported, it was calculated by the Fisher Exact Test on the basis of the number of observed and expected cases [11].

In addition to the pre-registered study protocol, we also extracted from each article the number of bladder cancer cases that would be expected under the null hypothesis that DEE had no association with bladder cancer. This number of expected cases served as a measure for the *amount of evidence* that each article contributed. The number of cases depends on the sample size and the prevalence of the disease. The expected number of cases in epidemiological research is often based on a population estimate and therefore more reliable than the observed number of cases that is based on the much smaller study sample, particularly in the case of rare diseases.

Finally, the variable *abstract reporting*—whether ‘bladder’ or ‘urinary’ or ‘transitional’ was mentioned in the abstract, title or keywords—was also extracted from the articles, and from the ‘keywords’ field of the online records to check if any relevant KeyWords Plus or MeSH terms had been added by the respective databases. This variable is dichotomous: articles with *incomplete abstract reporting* do not report bladder in their abstract, title, author keywords or database-specific keywords, whereas articles with *complete abstract reporting* do report bladder in any of these searchable fields. *Abstract reporting* served as a selection variable in our meta-analysis.

### Statistical analysis

We performed two main analyses on these data: (1) on the occurrence of incomplete abstract reporting and (2) on its impact on the pooled estimate.

First, we assessed the percentage of articles with incomplete abstract reporting, and the amount of evidence that these articles contain. We also performed a specific search strategy (Table 1) to check our assumption that the articles with incomplete abstract reporting would indeed be missed by a specific search strategy.

Secondly, we aimed to quantify the impact of incomplete abstract reporting on the pooled estimate. We ran two consecutive meta-analyses on the reported (unadjusted) effect sizes, one based on the full study sample, and one based on the subsample of articles with complete abstract reporting. The pooled estimate from the meta-analysis on the full study sample is considered to represent the true effect size, whereas the pooled estimate from the other meta-analysis, which is based on a limited and potentially biassed sample, might be an overestimation of the true effect size. For both meta-analyses, we used the inverse-variance method with random effects to pool the effects. As the reported effect sizes represent an incidence/risk ratio, they were log transformed first.

Our outcome variable is the *overestimation of the pooled estimate*. It was calculated by subtracting the (back-transformed) pooled estimate of the full sample meta-analysis from the (back-transformed) pooled estimate of the subsample meta-analysis.

In addition to our pre-registered data-analysis plan, we wanted to explore the impact of database-specific keywords on the detectability of the articles and on the overestimation of the pooled estimate. We therefore repeated the above analyses, but then without the database-specific keywords (thus only based on what the author had mentioned in the abstract, title or keywords).

## Results

We identified 28 articles with a cohort design on the association between DEE and bladder cancer (Fig. 1; Additional file 2: Text S2), representing a total of 5535 expected cases of bladder cancer.

### Occurrence of incomplete abstract reporting

**Fig. 2:**
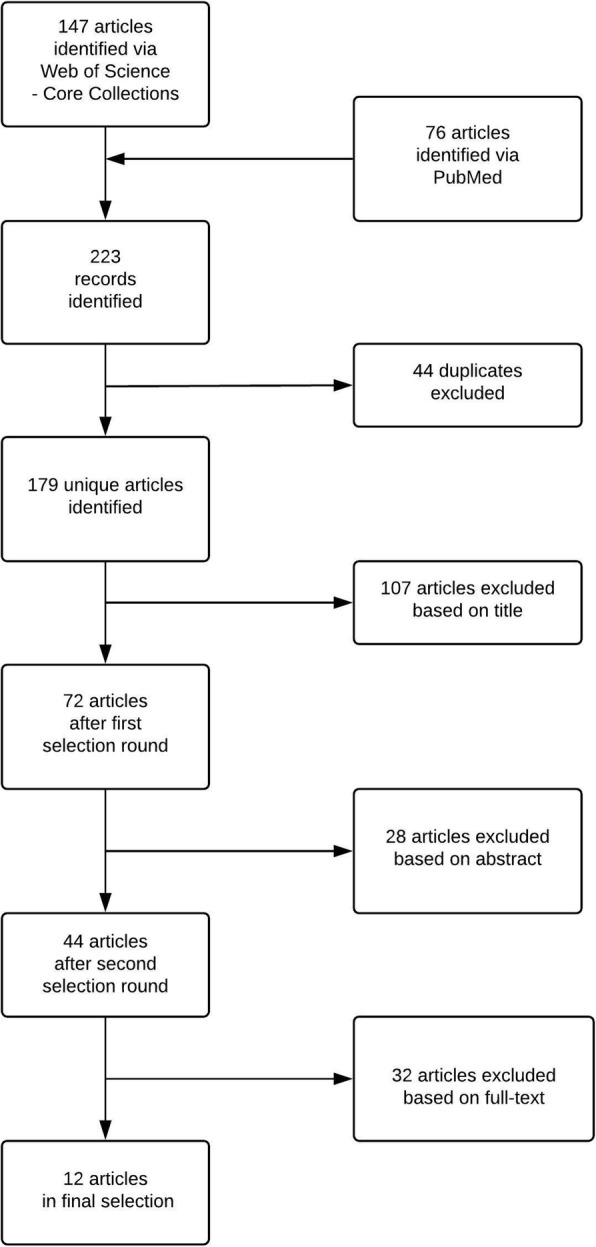
Flow diagram of article selection process (based on specific search query)

There were 16 articles with incomplete abstract reporting, representing 1056 expected bladder cancer cases (19%), and 12 articles with complete abstract reporting on bladder cancer. Applying the specific search (Table 1, Fig. 2) to WoSCC and PubMed confirmed our assumption that only the latter 12 articles were detectable by a search query restricted to bladder cancer (Table 2).

**Table 2:** Overview of the number of articles identified by different searches and with or without complete abstract reporting

	Total	Web of Science	PubMed
Number of articles in study population (based on broad search)	28	23	16
Number of articles retrieved by specific search*	12	11	4
Number of articles with complete abstract reporting*	12	11	4
Number of articles with complete abstract reporting (excluding database-added keywords)	9	8	4
Number of articles identified only on database-specific keywords	3	3	0

*These strategies were assumed and confirmed to identify exactly the same articles. ‘Articles with complete abstract reporting’ are articles that report ‘bladder’, ‘urinary’ or ‘transitional’ in their abstract, title or keywords

### Impact of incomplete abstract reporting

**Fig. 3:**
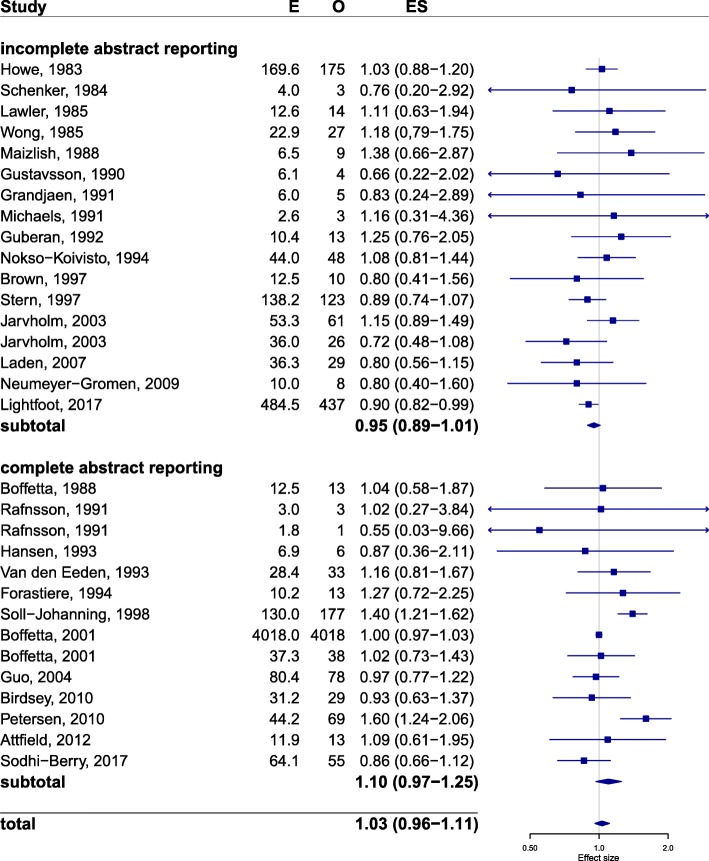
Forest plot based on the full sample. NB. The 28 articles in the full sample contain 31 unique associations between DEE and bladder cancer. I² = 43.6%, p = 0.006. E = Expected number of cases, O = Observed number of cases ES = Effect Size

The meta-analysis of the full study sample of 28 articles (Fig. 3) revealed a pooled estimate of 1.03 (95% confidence interval [95% CI] 0.96–1.11). The meta-analysis of the subsample of 12 articles with complete abstract reporting yielded a pooled estimate of 1.10 (95% CI 0.97–1.25). This suggests a 7-percentage point overestimation of the effect of DEE on bladder cancer if the articles with incomplete abstract reporting are missed (see also Additional file 3: Text S3).

### Impact of database-specific keywords

Three of the 12 detectable articles with complete abstract reporting (representing 43 expected cases) had not mentioned bladder in their abstract, title or author keywords. They could only be detected because ‘bladder’ had been added to the KeyWords Plus in Web of Science. MeSH terminology did not improve the detection of articles in this case study. Thus, without the database-specific keywords, only nine out of 28 articles would have been detected.

The meta-analysis of our subsample of nine articles with bladder reported in the abstract, title or author keywords yielded a pooled estimate of 1.12 (95% CI 0.97–1.30). The overestimation of the pooled estimate would have been 9 percentage points without the database-specific keywords.

## Discussion

We aimed to assess how many articles failed to mention relevant health outcomes in their abstract, title or keywords. Such articles are likely to be overlooked by a systematic review. In addition, we aimed to quantify the impact of overlooking these articles on the pooled estimate in a meta-analysis. In our case study on the association between diesel exhaust exposure and bladder cancer, we found that 12 out of 28 articles (57%) failed to report bladder cancer in any of the searchable fields. These articles represented 19% of all the evidence in this field. Neglecting this evidence led to an overestimation of seven percentage points of the pooled effect size.

Three of the articles could only be detected because of additional keywords provided in the Web of Science database. These keywords, that were automatically generated based on the titles in the reference lists of individual articles [12], do indeed seem to improve detection of relevant articles. Without the database-specific keywords, abstract reporting bias would have led to a 9-percentage point overestimation of the pooled estimate.

On the other hand, MeSH terms added to the MEDLINE/PubMed database did not improve detection in this case study. This is despite the fact that MeSH terms are based on full-texts that were manually scanned by human classifiers and that these classifiers were instructed to choose the most specific MeSH term possible [13]. The lack of improvement may be due to ‘the rule of three’. If more than three health outcomes are reported, then classifiers are allowed to choose the more general MeSH term (e.g. cancer), instead of specific MeSH terms for each separate health outcome. This rule is applied to the majority of articles in our study sample and probably to many other articles in the field of epidemiology as well.

We identified one meta-analysis, published in 2001, on the association between DEE and bladder cancer [14]. Unfortunately, no search strategy or database had been specified. The authors had included seven cohort studies on this topic, published between 1983 and 1998. We identified 18 cohort studies in the same period. Taking into account their inclusion criteria, they had missed seven cohort studies from our study sample, five of them with incomplete abstract reporting on bladder cancer. Even though they had checked the reference lists of already included articles, they still missed a large part of the literature, possibly due to incomplete abstract reporting.

We believe that this is the first study to introduce the concept of abstract reporting bias and its impact on systematic reviews. A recent study mapped 235 terms for different types of bias in the biomedical field [15], but abstract reporting bias was not among them. Neither was it mentioned in Song’s extensive review of dissemination biases [6], or in any other literature that we are aware of. Consequently, our study is also the first to propose a method to investigate the occurrence of this bias and its impact on systematic reviews. In our case study on the association between DEE and bladder cancer, we further showed that the failure to report bladder in the abstract, title or keywords can indeed lead to the neglect of a substantial part of the available evidence.

Our study has some limitations. For instance, we may have missed some relevant articles in the search output of our broad search. Even though we were very conservative in excluding articles based on their title or abstract, it is possible that we excluded articles that mentioned only one cancer outcome in the abstract while reporting multiple cancer outcomes in the full-text. If indeed we failed to identify relevant articles due to incomplete abstract reporting, then bias due to incomplete abstract reporting is likely to be larger. A related limitation has to do with the exposure variable. Even though our broad search strategy involved any type of cancer, it was still quite restrictive with regard to our exposure variable (diesel exhaust exposure). If we had set up an even broader search strategy, more relevant articles might have been retrieved. Finally, we need to emphasise that the pooled estimate of the meta-analysis on our complete study sample is not necessarily the true estimate of the association between DEE and bladder cancer. After all, only cohort studies were included, the search strategy could have been even broader, and the meta-analyses were based on unadjusted effect sizes. Nevertheless, we believe that our analysis is sufficient for our research aim: to investigate the occurrence and impact of abstract reporting bias. Similarly, we did not assess the risk of bias of the included articles in our study. We believe this is not relevant in a proof-of-concept study.

Despite the limitations, we believe that our study has shown the harmful potential of abstract reporting bias. At the same time, we want to stress that this is not the fault of the authors that wrote the articles. On the contrary, they made efficient use of their study investment by measuring multiple health outcomes at once, and it seems that they reported all of them regardless of their direction or statistical significance. If they indeed did, they would have acted in line with current publication guidelines [16, 17]. Our study shows that even if all results are conscientiously reported in the full-text of articles, there would still be a problem if not all these results can be found. Especially now that the number of publications is growing exponentially, it becomes harder to locate them efficiently.

When would abstract reporting bias be most likely to occur? We believe that it is not likely to occur if a certain association is generally believed to exist, such as in the case of smoking and lung cancer. If a study would find negative results for such association, it would be considered surprising and as such worthy to be reported in the abstract. When an association is the topic of dispute, such as in the case of DEE and lung cancer [18], abstract reporting bias is equally unlikely to occur. These results are considered of interest regardless of their outcome and therefore likely to reach the abstract, title or keywords. However, for health outcomes for which it is less obvious that they might be associated with the exposure studied, the preference to focus on positive findings in the abstract comes into play.

So what can we do about this? One solution would be that search engines improve their functionality by allowing full-text search. Google Scholar has already implemented this functionality [19]. However, their search algorithm is not transparent and has limited functionality for the use of operators, so we recommend against relying on their search engine for a systematic search. However, applying a specific search (i.e. *diesel bladder cancer*) in Google Scholar shows how the output explodes when searching within full-texts: from 12 for searching within the title only to 9740 for searching in the full-text. Only a small fraction of the output should have been included in our study, and with the exponential and on-going increase in the number of publications, this fraction will only become smaller. A more efficient way is needed to detect relevant articles.

Rather than implementing full-text search functionality, we propose to report all outcomes and exposure variables in the searchable fields like abstract or keywords. Reporting all these variables in the abstract is often not feasible nor desirable. After all, an abstract is supposed to give a summary. However, the number of keywords could easily be extended. Many journals currently maintain a restriction on the maximum number of author-provided keywords, with often no more than 5 or 6 keywords allowed. If journals would drop this restriction, and authors report each studied exposure and outcome variable as a keyword, this would profoundly increase the detection of these articles. We believe that this solution is easy to implement.

In the long run, however, we plead for more specialised search fields. The current publication system is largely based on old-fashioned paper logic, but we now live in the digital era that allows for much more flexibility. We believe that having separate fields for predictors, outcomes variables, confounders, study design and type of subjects would greatly improve both the sensitivity and the specificity of search results.

This may take time to implement, and it will not change the already published literature. For researchers who currently want to conduct a systematic review, we suggest to apply a broad search strategy [20] and to always check the full-text of an article if the title or abstract suggests that multiple associations might have been tested. Such search strategy is more laborious, but it limits the chances of missing relevant literature.

## Conclusions

We found that more than half of the articles, containing almost 20% of the available evidence, suffered from incomplete abstract reporting and were difficult to identify by a systematic search. This led to an overestimation of 7 percentage points of the pooled effect size. We urge journal editors to drop the restriction on the number of keywords, in order to enhance the likelihood for articles to be found.

## Additional files

Additional file 1: Text S1. Protocol deviations (DOCX 208 kb)

Additional file 2: Text S2. Reference list of articles included in our study sample. (DOCX 346 kb)

Additional file 3: Text S3. Meta-analysis and meta-regression. (DOCX 101 kb)

## Data Availability

The protocol, including the data-analysis plan, and the data set of this study are available upon request in the DataVerse repository, http://hdl.handle.net/10411/5798UX, or by sending an email to b.duyx@maastrichtuniversity.nl.

## Abbreviations

MeSH: Medical Subject Headings
DEE: Diesel exhaust exposure
WoSCC: Web of Science Core Collection
95% CI: 95% confidence interval
ICD: International Classification of Diseases
