# Supplementary material for: The strong focus on positive results in abstracts may cause bias in systematic reviews: a case study on abstract reporting bias
Source: Syst Rev. 2019 Jul 17;8:174. doi: 10.1186/s13643-019-1082-9 (PMC6637611; doi:10.1186/s13643-019-1082-9)
Supplement: Supplementary file 1 [file 13643_2019_1082_MOESM1_ESM.docx]

Abstract Bias -- Supplement

Text S1. Protocol deviations

Because we did not retrieve enough articles from Web of Science, we extended our search to PubMed (and not just to Medline as was written in the protocol, but Medline is included in PubMed).

The word 'transitional' was added to the specific search strategy (and to the data-extraction of the variable *abstract reporting*) to identify transitional cell carcinoma, a type of carcinoma that often appears at the bladder site. None of the articles in our sample had included this word, so it did not change our results.

The chi-square analysis, to analyse the *occurrence* of abstract reporting bias, was skipped because only two supportive articles were identified (being statistically significant in the expected direction).

Similarly, the analysis on the difference in selective abstract reporting before and after publication of the IARC report was skipped, because only two articles after 2013 were found. (We wondered if this report would have made authors more aware of the potential link between diesel exhaust exposure and bladder cancer, and hence report it more often in their abstract, regardless of study outcome.)

The sensitivity and specificity analysis of the broad and the specific search queries, as mentioned in the protocol, were skipped. However, flow diagrams of both searchers were presented, so the sensitivity and specificity of both searches can be calculated.

Extra meta-analyses and meta-regression were added to assess the impact of database-specific keywords. Therefore, the analyses of the effect of selective abstract reporting on the pooled estimate were repeated, but then based on the abstract, title and author-provided keywords only.

A measure for the *amount of evidence* was added. After all, articles with a larger sample size and more cases of bladder cancer, provide more evidence for or against the tested hypothesis than articles with a small sample size do. We based our measure for the amount of evidence on the number of expected bladder cancer cases.

For some included studies, the confidence intervals, standard errors, or number of expected bladder cancer cases had to be calculated by hand based on the reported results. The standard error was calculated by dividing the range of the log-transformed confidence interval by 3.92. If not reported, we either used the confidence interval reported in a meta-analysis (1), or used the Fisher exact test results on http://www.openepi.com/SMR/SMR.htm. If not reported, the expected number of bladder cancer cases was found by dividing the observed number of cases by the risk point estimate. In one case (reference 11 in Text S2), in which neither the expected nor the observed number of cases was reported, we found it by trial and error on http://www.openepi.com/SMR/SMR.htm, by filling in different combinations of numbers until we replicated the point estimate and confidence interval that were reported in the article.

1. Boffetta P, Silverman DT. A meta-analysis of bladder cancer and diesel exhaust exposure. Epidemiology. 2001;12(1):125-30.
