# Supplementary material for: The strong focus on positive results in abstracts may cause bias in systematic reviews: a case study on abstract reporting bias
Source: Syst Rev. 2019 Jul 17;8:174. doi: 10.1186/s13643-019-1082-9 (PMC6637611; doi:10.1186/s13643-019-1082-9)
Supplement: Supplementary file 2 [file 13643_2019_1082_MOESM2_ESM.docx]

Text S2. Reference list of articles in our study sample

1. Attfield MD, Schleiff PL, Lubin JH, Blair A, Stewart PA, Vermeulen R, et al. The diesel exhaust in miners study: A cohort mortality study with emphasis on lung cancer. JNCI- Natl Cancer Inst. 2012;104(11):869-83.

2. Birdsey J, Alterman T, Li J, Petersen MR, Sestito J. Mortality among members of a truck driver trade association. Aaohn J. 2010;58(11):473-80.

3. Boffetta P, Dosemeci M, Gridley G, Bath H, Moradi T, Silverman D. Occupational exposure to diesel engine emissions and risk of cancer in swedish men and women. Cancer Causes Control. 2001;12(4):365-74.

4. Boffetta P, Stellman SD, Garfinkel L. Diesel exhaust exposure and mortality among males in the american-cancer-society prospective-study. Am J Ind Med. 1988;14(4):403-15.

5. Brown AM, Christie D, Taylor R, Seccombe MA, Coates MS. The occurrence of cancer in a cohort of new south wales coal miners. Aust N Z Publ Health. 1997;21(1):29-32.

6. Forastiere F, Perucci CA, Dipietro A, Miceli M, Rapiti E, Bargagli A, et al. Mortality among urban policemen in rome. Am J Ind Med. 1994;26(6):785-98.

7. Grandjean P, Andersen O. Lung-cancer in filling station attendants. Am J Ind Med. 1991;20(6):763-8.

8. Guberan E, Usel M, Raymond L, Bolay J, Fioretta G, Puissant J. Increased risk for lung-cancer and for cancer of the gastrointestinal-tract among geneva professional drivers. British Journal of Industrial Medicine. 1992;49(5):337-44.

9. Guo J, Kauppinen T, Kyyronen P, Heikkila P, Lindbohm ML, Pukkala F. Risk of esophageal, ovarian, testicular, kidney and bladder cancers and leukemia among finnish workers exposed to diesel or gasoline engine exhaust. Int J Cancer. 2004;111(2):286-92.

10. Gustavsson P, Plato N, Lidstrom EB, Hogstedt C. Lung-cancer and exposure to diesel exhaust among bus garage workers. Scand J Work Environ Health. 1990;16(5):348-54.

11. Hansen ES. A follow-up-study on the mortality of truck drivers. Am J Ind Med. 1993;23(5):811-21.

12. Howe GR, Fraser D, Lindsay J, Presnal B, Yu SZ. Cancer mortality (1965-77) in relation to diesel fume and coal exposure in a cohort of retired railway workers. J Natl Cancer Inst. 1983;70(6):1015-9.

13. Jarvholm B, Silverman D. Lung cancer in heavy equipment operators and truck drivers with diesel exhaust exposure in the construction industry. Occup Environ Med. 2003;60(7):516-20.

14. Laden F, Hart JE, Smith TJ, Davis ME, Garshick E. Cause-specific mortality in the unionized us trucking industry. Environ Health Perspect. 2007;115(8):1192-6.

15. Lawler AB, Mandel JS, Schuman LM, Lubin JH. A retrospective cohort mortality study of iron ore (hematite) miners in minnesota. Journal of occupational medicine : official publication of the Industrial Medical Association. 1985;27(7):507-17.

16. Lightfoot NE, Berriault CJ, Seilkop SK, Conard BR. Nonrespiratory mortality and cancer incidence in a cohort of canadian nickel workers. Arch Environ Occup Health. 2017;72(4):187-203.

17. Maizlish N, Beaumont J, Singleton J. Mortality among california highway workers. Am J Ind Med. 1988;13(3):363-79.

18. Michaels D, Zoloth SR. Mortality among urban bus drivers. Int J Epidemiol. 1991;20(2):399-404.

19. Neumeyer-Gromen A, Razum O, Kersten N, Seidler A, Zeeb H. Diesel motor emissions and lung cancer mortality-results of the second follow-up of a cohort study in potash miners. Int J Cancer. 2009;124(8):1900-6.

20. Noksokoivisto P, Pukkala E. Past exposure to asbestos and combustion products and incidence of cancer among finnish locomotive drivers. Occup Environ Med. 1994;51(5):330-4.

21. Petersen A, Hansen J, Olsen JH, Netterstrom B. Cancer morbidity among danish male urban bus drivers: A historical cohort study. Am J Ind Med. 2010;53(7):757-61.

22. Rafnsson V, Gunnarsdottir H. Mortality among professional drivers. Scand J Work Environ Health. 1991;17(5):312-7.

23. Schenker MB, Smith T, Munoz A, Woskie S, Speizer FE. Diesel exposure and mortality among railway workers: Results of a pilot study. Br J Ind Med. 1984;41(3):320-7.

24. Sodhi-Berry N, Reid A, Fritschi L, Musk AW, Vermeulen R, de Klerk N, et al. Cancer incidence in the western australian mining industry (1996-2013). Cancer Epidemiol. 2017;49:8-18.

25. Soll-Johanning H, Bach E, Olsen JH, Tuchsen F. Cancer incidence in urban bus drivers and tramway employees: A retrospective cohort study. Occup Environ Med. 1998;55(9):594-8.

26. Stern F, HaringSweeney M. Proportionate mortality among unionized construction operating engineers. Am J Ind Med. 1997;32(1):51-65.

27. Vandeneeden SK, Friedman GD. Exposure to engine exhaust and risk of subsequent cancer. J Occup Environ Med. 1993;35(3):307-11.

28. Wong O, Morgan RW, Kheifets L, Larson SR, Whorton MD. Mortality among members of a heavy construction equipment operators union with potential exposure to diesel exhaust emissions. Br J Ind Med. 1985;42(7):435-48.
