# Supplementary material for: The strong focus on positive results in abstracts may cause bias in systematic reviews: a case study on abstract reporting bias
Source: Syst Rev. 2019 Jul 17;8:174. doi: 10.1186/s13643-019-1082-9 (PMC6637611; doi:10.1186/s13643-019-1082-9)
Supplement: Supplementary file 3 [file 13643_2019_1082_MOESM3_ESM.docx]

Text S3. Meta-analysis and meta-regression

Our study sample consisted of 28 articles. Some had reported multiple tests on diesel exhaust exposure – bladder cancer, on separate, non-overlapping groups of subjects. In total, 31 effect size measures were included in the meta-analysis. We used Stata 13.1 to perform the meta-analyses and meta-regressions described below. Meta-regression was used to statistically test whether the articles with complete abstract reporting yielded a different pooled estimated compared to the articles with selective abstract reporting.

The meta-analysis of the full study sample of 28 publications revealed a pooled estimate of 1.03 (95% confidence interval [CI] 0.96 – 1.12). The meta-analysis of the subsample with complete abstract reporting yielded a pooled estimate of 1.10 (CI 0.97 – 1.25). Meta-regression showed no statistically significant effect of selective abstract reporting on the pooled estimate (OR 1.14, CI 0.97-1.34).

The meta-analysis of our subsample with complete abstract reporting, but without the articles that could only by identified because of the databases-specific keywords, yielded a pooled estimate of 1.12 (CI 0.97 – 1.30). Meta-regression showed a marginally significant effect of selective abstract reporting on the pooled estimate (OR 1.17, CI 0.99-1.37).
